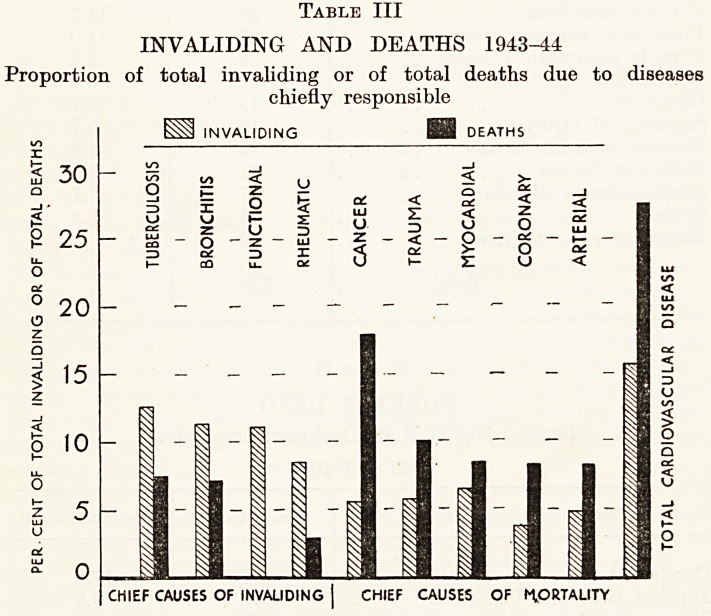# Symptomless Hyperpiesia in Dockyard Workers

**Published:** 1946

**Authors:** J. M. Naish

**Affiliations:** Medical Registrar, Bristol Royal Infirmary


### SYMPTOMLESS HYPERPIESIA IN DOCKYARD WORKERS

BY

J. M. Naish, M.A., M.B., B.Ch., M.R.C.P.
*Medical Registrar, Bristol Royal Infirmary.*

---

While working in one of H.M. Dockyards, I was impressed on the one hand by the healthy appearance and good expectation of life in those who were for physical reasons virtually invalids, or for mental reasons unemployable ; and on the other hand by the severe pathological processes which came to light on examination of those good workers who "had never had a day's illness in their lives," who would undoubtedly go on working well, metaphorically speaking, until they dropped. To put the matter more briefly—the eminently insurable were often unemployable, and the eminently employable were often uninsurable. Too frequently a man carrying the burden of a fatal prognosis is denied the consolation of dying in harness, and industry is deprived of a useful servant. As an example, one has only to consider the vast numbers of hyperpietic subjects who are forbidden useful work simply because they have a high blood pressure ; worse, many of them develop a doctor-made neurosis which thereafter effectively prevents them from working even if they are allowed to do so.

Symptomless hyperpiesia as it occurs in working men at the age of 60 has been the subject of this small study. My object has been to try and show first whether hyperpietics, who are known to carry a short expectation of life,[1] are particularly prone to chronic ill health or to die within a short time after the diagnosis has been made; secondly, to determine the incidence of hyperpiesia in working men between the ages of 60 and 65 ; and thirdly to try and assess the effect of hyperpiesia upon a man's usefulness in industry. To get an idea of the effect of hyperpiesia on health, it was necessary to consider the effect of cardiovascular disease in general on life and industrial efficiency, since hyperpiesia is known to be the greatest single factor in the production of cardiovascular disease.[2] To this end, I scrutinised the health records of H.M. Dockyard for two successive years.

During these two years, 1943 and 1944, the average number of employees at H.M. Dockyard was 20,995. In this period 335 persons were invalided (Table I), and 314 persons died (Table II) while still

TABLE I

WORKERS INVALIDED

(from among 20,995 H.M. Dockyard employees)

1943 and 1944

| Disease or Disease Group | Total No. of Cases | Per cent. of Total |
|---|---|---|
| Pulmonary tuberculosis .. .. | 43 | 12.5 |
| Chronic bronchitis .. .. .. | 38 | 11.3 |
| Functional nervous disorders .. | 37 | 11.0 |
| Chronic rheumatic diseases .. .. | 28 | 8.4 |
| Myocardial degeneration .. .. | 22 | 6.6 |
| Carcinoma .. .. .. .. | 19 | 5.7 |
| Sequelae of injuries .. .. .. | 19 | 5.7 |
| Cerebral arterial disease .. .. | 16 | 4.8 |
| Coronary disease .. .. .. | 11 | 3.8 |
| Genito-urinary disease .. .. | 7 | 2.1 |
| Syphilis .. .. .. .. .. | 6 | 1.8 |
| Miscellaneous conditions .. .. | 89 | |
| Total .. | 335 | |

TABLE II

WORKERS DEAD

(among 20,995 H.M. Dockyard employees)

1943 and 1944

| Disease or Disease Group | No. of Cases | Per cent. of Total |
|---|---|---|
| Carcinoma .. .. .. .. | 56 | 17.8 |
| Injuries (war and industrial) .. | 31 | 9.9 |
| Myocardial degeneration .. .. | 27 | 8.5 |
| Coronary disease .. .. .. | 25 | 8.0 |
| Cerebral arterial disease .. .. | 25 | 8.0 |
| Pneumonia .. .. .. .. | 25 | 8.0 |
| Pulmonary tuberculosis .. .. | 23 | 7.3 |
| Bronchitis .. .. .. .. | 22 | 7.0 |
| Genito-urinary disease .. .. | 13 | 4.1 |
| Rheumatic heart disease .. .. | 9 | 2.9 |
| Syphilis .. .. .. .. .. | 7 | 2.2 |
| Miscellaneous conditions .. .. | 51 | |
| Total .. .. | 314 | |

in employ at the dockyard. Generally speaking a man was not invalided until he had been off work for six months. There is no direct evidence as to the ultimate fate of the 335 persons who were invalided, but from a consideration of the diseases from which they suffered, it would be fair to assume that a large proportion survived for a considerable time. These figures, then, should give an approximate picture of the chief causes of rapid death on the one hand, and chronic invalidism on the other : Table III shews these in graphic form.

TABLE III

INVALIDING AND DEATHS 1943–44

Proportion of total invaliding or of total deaths due to diseases chiefly responsible

In the present series cardiovascular disease caused 27.4 per cent. of all deaths while in employment. The killing power of cardiovascular disease is well known. As a cause of chronic invalidism it is comparatively less important ; only 15 per cent. of workers invalided suffered chiefly from cardiovascular disease. It would be correct to state that cardiovascular disease caused a high proportion of sudden deaths, and deaths after a comparatively short illness, and a smaller proportion of prolonged illness leading to eventual death. It has been estimated that hypertension is the greatest single factor in the aetiology of coronary thrombosis,[2] and is more frequently associated with cardiovascular disease than any other finding. It is therefore not surprising that hyperpiesia, which is one of the most common measurable abnormalities of the cardiovascular system, should prove such a true guide to the life expectation of any given individual, and is used so widely as such by the insurance companies.

On the other hand, it is well known that hyperpiesia exists in a large proportion of the population without producing symptoms. This is the so-called benign hypertension or hyperpiesia of Allbutt.[3] To estimate the frequency of this condition, large surveys of the general population have been carried out from time to time, using the data collected by insurance companies. It has been suggested that no less than one-fifth of the total population is either hypertensive or prehypertensive.[2] In order to try and find out how often hyperpiesia is symptomless amongst the older subjects of the disease, I examined nearly 200 working men between the ages of 60 and 65, with particular reference to their blood-pressures, the state of their arteries and heart, the presence or absence of symptoms, and their fitness for continued employment.

Before there can be any estimation of the frequency of hyperpiesia an attempt has to be made to determine what is a normal figure for the blood pressure. In this attempt one is hindered by the recognised inaccuracies in the measurement of intra-arterial tension by a sphygmomanometer. There are significant differences between the intra-arterial tension measured by the direct and the sphygmomanometric methods[5]; the sphygmomanometric reading is affected by the girth of the arm, and errors of $\pm$ 10 mm. Hg. or more have been detected in nearly all subjects, due to undetermined factors. Apart from the errors of the sphygmomanometer, it is known that there are true variations in an individual's blood pressure.[6] The pressor effect of the examining physician is a fairly constant factor no matter what the environment of the consulting room,[7, 8] but as it presumably exists for all doctors it can reasonably be ignored. Variations of up to 80 mm. Hg. in the systolic pressures and 35 mm. Hg. in the diastolic have been recorded in the same individual. These variations are, however, only of this order in the hypertensive or pre-hypertensive person[9, 10]: in normal people the variations are less. Nevertheless, it seems that a single sphygmomanometric estimation of blood pressure cannot be taken as an accurate measurement of a man's normal intra-arterial tension. A variation of at least 50 mm. Hg. systolic, and 25 mm. Hg. diastolic, has to be expected due to the physical inaccuracies of the method, and the varying tone of the individual's arteries. In their large survey, Robinson and Brucer[2] conclude that 120–90 mm. Hg. is the normal systolic and 60–80 mm. Hg. the normal diastolic range. These figures are lower than those usually accepted: the usual practice being to consider as abnormal systolic pressures over 140 mm. Hg. and diastolic pressures over 90 mm. Hg. But Robinson and Brucer's figures of the normal are obtained by taking the mean of their sample when those with pressures over 140 mm. Hg. systolic and 90 mm. Hg. diastolic, who are considered to be hypertensive, have been ruthlessly excluded from that sample. Rightly they insist that an attempt to find a *normal* blood pressure should not be confused with finding an *average* blood pressure for the whole population, hypertensives included. Regarding all the above factors, the known physical inaccuracies of the sphygmomanometric measurements of blood pressure, the known variations of that pressure in a given individual, and the fact that in the present series only a single reading was taken, it was considered safest to take as abnormal only those systolic readings which are 50 mm. Hg., and those diastolic readings which are 25 mm. Hg., above the highest average normal figure. Thus systolic pressures over 170 mm. Hg. and diastolic blood pressures over 105 mm. Hg. are taken to indicate true hyperpiesia.

It is a well recognised fact, and especially since the days of Clifford Allbutt, that arteriosclerosis and gross arterial disease can exist almost as frequently without as with true hyperpiesia.[1, 3, 4] It is known that rigidity of peripheral arteries due to arteriosclerotic thickening causes a general loss of elasticity in the arterial bed, and that consequently the systolic stroke of the heart produces an abrupt and rapid transmission of the pulse wave ; where, therefore, the arteries are abnormally thickened and rigid, high systolic and normal diastolic pressures may be met with. In the present series there is a definite group of " systolic hypertensives " in whom was also found evidence of thickening, rigidity, and tortuosity of the medium-sized arteries : these are not considered to be suffering from the same disease process as the true hyperpietics, and are therefore shown separately. Thus those with systolic blood pressures of over 170 mm. Hg. but diastolic pressures below 105 mm. Hg. are shown as suffering from " systolic hyperpiesia."

Table IV gives a conservative picture of the incidence of true

TABLE IV

CARDIOVASCULAR ABNORMALITIES IN 188 MEN AT WORK AGED 60–65

| Disease | No. of Cases | Per cent. of Total |
|---|---|---|
| Hyperpiesia . . . . . . . . | 38 | 20.2 |
| " Systolic hyperpiesia " . . . . | 55 | 29.3 |
| Other cardiovascular diseases . . | 6 | 3.2 |
| Normal blood pressures . . . . | 89 | 47.3 |

hyperpiesia, because, owing to the danger of including normal people with fluctuating blood-pressures in this group, the level above which blood pressures are considered to be abnormal has been set very high. Only if repeated examinations of each individual[6] are made under standard conditions can this level be set lower. But it is certain that benign hyperpiesia is considerably commoner than is generally realised. Thirty out of the 38 cases suffering from hyperpiesia had blood pressures over the 200/105 mark ; one had blood pressure 298/152 without symptoms or other abnormal physical signs ; and although in ten of them there was clinical evidence of enlargement of the left ventricle, few of them complained of symptoms referable to the state of their circulation. Nearly all the men who complained of a feeling of fullness in the head, dizziness, headaches, generalised aching and fatigue, symptoms usually said to be due to hyperpiesia, had blood pressures considered to be within the normal range, and the three hyperpietics who complained thus all gave definite histories of previous nervous instability (Table V). This is in accordance with previous studies which have

TABLE V

Seventeen working men who complained of dizziness, throbbing, tinnitus, or of dyspnoea on exertion

| Symptoms and Disposal | Hyperpiesia | Systolic Hyperpiesia | Normal B.P.'s |
|---|---|---|---|
| Dizziness, Throbbing sensations, Tinnitus.. | 3 All with associated neuroses | 1 | 4 |
| Dyspnoea on exertion | 5 | 0 | 4 Emphysema, Chronic bronchitis. Effort syndrome |
| Number invalided .. | 5 Two where primary disability was from other diseases | 1 With other disease | 3 |

shown that the " early " symptoms of hyperpiesia are identical with those of psycho-neurosis.[11] In only three out of the five cases of hyperpiesia who were found unfit for further work was that unfitness due to the indirect effects of hyperpiesia ; one of these men with blood pressure 182/105 suffered from dyspnoea on exertion and enlarged heart, another had dyspnoea and auricular fibrillation. The remaining cases were invalided chiefly on account of associated severe and disabling conditions of which they complained, such as chronic fibrositis, varicose ulcers, severe haemorrhoids, or neuroses.

"Other cardiovascular diseases" includes one case each of malignant hypertension, aortic incompetence, aortic aneurysm, probable patent interventricular septum, and two cases of rheumatic mitral disease. The man suffering from malignant hypertension whose blood pressure was 300+/165, and who had papilloedema, retinal haemorrhages, and albuminuria, was invalided. The other five were in fair general condition, had no complaints, and were considered fit for work.

Two inferences may be drawn from this small survey. The first is that hyperpiesia is a very common disorder, commoner perhaps than the figure of 20.2% would indicate because of the conservative delineation of the abnormal used, and because the sample is composed only of those living and fit for work at the age of 60. Hyperpietics tend to die earlier than their fellows, so 20.2% is no indication of the true attack-rate of the disease. In the same way Robinson and Brucer's[2] estimate that one fifth of the population is hypertensive or pre-hypertensive refers only to the living population ; there are fewer hypertensives than non-hypertensives alive between the ages of 50 and 80, so theirs is probably an under-estimate of the true incidence of the disease as it attacks humanity. It would no doubt be fair to state that nearly one man in four who reaches adult life will ultimately suffer from hyperpiesia. The second inference is that hyperpiesia is largely symptomless. This is perhaps generally accepted but not appreciated. A man can get along well with a very high blood pressure ; his heart may hypertrophy, it is true, but for many years he seems quite unaware of it. Amongst working men of all ages dyspnoea on exertion is met with much more frequently in chronic bronchitics. As for the other symptoms usually attributed to hyperpiesia—headaches, noises in the ears, dizziness, flushing, fatigue—these are met with just as frequently amongst those with normal blood pressures, and where these symptoms do occur in hyperpietics they certainly do not increase in severity as the blood pressure rises.[12] It is possible that this idea has arisen from the frequent association of mild degrees of hyperpiesia with the female menopause[13] : thus the symptoms of the menopause are attributed to hyperpiesia, though the hyperpiesia is often transient and may be due to the menopausal changes themselves.

When, however, hyperpiesia by itself is considered as a bar to employment there is danger of a mistake. Behneman[14] expresses the correct attitude: "If . . . (he) is found to have heart disease or circulatory hypertension, his acceptance or rejection (*in industry*) should depend solely on whether the work he is to do will increase that disorder, or whether the disorder will reduce his efficiency in that job."

These questions, then, have to be answered in considering the hyperpietic in relation to industry: will his work increase that disorder, or will the disorder affect his usefulness in industry by his inefficiency or the discontinuity of his work? [14, 15] Though I have stated that the hyperpietic "goes along very well" with his high blood pressure, the question as to whether his labour is doing him any harm has not been answered. The ground here is very uncertain. An experiment on the grand scale would be necessary to obtain evidence on this point. So far, though it is indisputable that a failing heart must not be overworked, there is no direct evidence to suggest that physical labour within the range of comfort harms the well hypertrophied heart of an hyperpietic. Figures comparing the relative incidence of cerebral haemorrhage, coronary thrombosis, and heart failure in sedentary and manual workers might be of some value. Hoskins[16] found 73% of sedentary workers in his series of cases of coronary thrombosis. Certainly the large proportion of symptomless hyperpietics detected in the present series would indicate no undue mortality from the condition in manual labourers. But even if labour was proved to be slightly harmful to hyperpietics, would it be right to deprive an elderly man of his work, interest, and livelihood when he himself feels perfectly fit? Too often, if a man is told that he has a "high blood pressure," his whole life is coloured by the ingrained horror of that painfully descriptive diagnosis: he develops unpleasant symptoms, chiefly of neurotic origin, and becomes a burden to himself and his family. All are compelled to bow before the idol of the sphygmomanometer. If it is a short life for a hyperpietic, let it be a merry one; nothing can be worse than to restrict a man's activities because he has a high blood pressure, if that means that he walks the rest of his life in fear. As for the so-called "management of the hyperpietic" and the various remedies designed to bring about a reduction of the blood pressure, I can only think of these as essentially useless and mentally harmful. A strong case can be made out for keeping the hyperpietic in ignorance of his accidentally discovered condition, and even against limiting his exertions upon some false pretext.

Does hyperpiesia affect a man's usefulness in industry? Almost all the hyperpietics in the present series admitted to no symptoms or reduction in efficiency, and were quite unaware that they had high blood pressures. Hyperpiesia by itself without a failing heart does not reduce a man's efficiency in any way that can be determined.

Lynch[17] considers that even cardiac patients with definite disabilities can work well in at least 25% of all jobs in industry, and that disabled workers are generally more careful and conscientious than their fellows. With regard to continuity of work, there is no evidence to suggest that hyperpietics are more prone to minor ailments or illnesses than others. When and if a hyperpietic develops a non-fatal coronary thrombosis, auricular fibrillation, or signs of heart failure with a regular rhythm, a different assessment must be made. But generally speaking, when their hearts fail or their cerebral arteries give way they are approaching their end. Compared with sufferers from chronic bronchitis, the hyperpietics render very steady service until their last illness.

In this paper I have attempted to put forward a point of view upon the well-established facts of medical knowledge rather than to present new facts themselves. It is inevitable that conceptions should change as the focus of medical attention shifts from the consulting room to the workshop, as the physician's view is widened to include the welfare of a trade or the State itself as well as that of the patient. Thus rheumatic and mental diseases, though frequently seen in the clinic and consulting room, are more readily known for the burden they are upon society when they are seen from the factory and the workshop ; and hyperpiesia, so common also and so dramatic in its effects upon the individual, appears of much more importance in the clinic than it does in the factory.

### SUMMARY.

The chief causes of death and invalidism amongst workers in a dockyard employing 20,000 men and women during 1943 and 1944 are discussed.

One hundred and eighty-eight working men between the ages of 60 and 65 were examined with particular reference to their blood pressures and cardiovascular systems. Using a conservative standard of abnormal blood pressure, over 20 per cent. of those examined were hyperpietic.

The majority of these 20 per cent. were symptomless, and the incidence of the so-called " early symptoms " of hyperpiesia was as great in the non-hyperpietic subjects.

A plea is made for keeping hyperpietics in ignorance of their blood-pressures.

REFERENCES.

1 Daley, R., Ungerleider, H., and Gubner, R., *Jour. Amer. Med. Assoc.*, 1943, cxxi. 383.

2 Robinson, S., and Brucer, M., *Arch. Int. Med.*, 1939, lxiv. 409; 1940, lxvi. 393.

3 Allbutt, T. C., " *Arteriosclerosis, a Summary View.*" London, 1925.

4 Allbutt, T. C., *Diseases of the Arteries, including Angina Pectoris.* London, 1915, Vol. II. 81.

5 Ragan, C., and Bordley III. J., *Bull., Johns Hopkins Hosp.*, 1941, lxix. 504.

6 Alam, G., and Smirk, F., *Brit. Heart Jour.*, 1943, v. 152.

7 Ayman, D., and Goldshine, A., *Amer. Jour. Med. Sci.*, 1940, cc. 465.

8 Ayman, D., and Goldshine, A., *Amer. Jour. Med. Sci.*, 1941, cci. 157.

9 Mountain, G. E. and Allen, E. V., *Proc. Staff Meet., Mayo Clin.*, 1941, xvi. 260.

10 Hines, E. A., *Jour. Amer. Med. Assoc.*, 1939, cxii. 1016.

11 Ayman, D., and Pratt, J., *Arch. Int. Med.*, 1931, xlvii. 675.

12 Halls, Dally J., *Brit. Encyclo. Med. Practice*, Vol II, 511–518.

13 Riseman, J., and Weiss, S., *Amer. Jour. Med. Sci.*, 1930, clxxx. 47.

14 Behneman, H. M. F., *Jour. Amer. Med. Assoc.*, 1941, cxvi. 209.

15 Carlisle, J., and Gibson, A., *Indust. Med.*, October, 1944, xiii. 783.

16 Hoskins, T. J., *The Practitioner*, 1944, cliii. 136.

17 Lynch, D. L., *Jour. Amer. Med. Assoc.*, 1941, cxvi. 1380.

## Figures and Tables

**Table III f1:**